# Dexmedetomidine postconditioning attenuates myocardial ischemia/reperfusion injury by activating the Nrf2/Sirt3/SOD2 signaling pathway in the rats

**DOI:** 10.1080/13510002.2022.2158526

**Published:** 2023-02-04

**Authors:** Bin Hu, Tian Tian, Xin-Tao Li, Pei-Pei Hao, Wei-Chao Liu, Ying-Gui Chen, Tian-Yu Jiang, Pei-Shan Chen, Yi Cheng, Fu-Shan Xue

**Affiliations:** Department of Anesthesiology, Beijing Friendship Hospital, Capital Medical University, Beijing, People’s Republic of China

**Keywords:** Dexmedetomidine, myocardial ischemia/reperfusion injury, oxidative stress, apoptosis, Nrf2/Sirt3/SOD2 signaling pathway

## Abstract

**Objectives:**

To observe the protective effects of dexmedetomidine (Dex) postconditioning on myocardial ischemia/reperfusion injury (IRI) and to explore its potential molecular mechanisms.

**Methods:**

One-hundred forty-seven male Sprague-Dawley rats were randomly divided into five groups receiving the different treatments: Sham, ischemia/reperfusion (I/R), Dex, Brusatol, Dex + Brusatol. By the *in vivo* rat model of myocardial IRI, cardioprotective effects of Dex postconditioning were evaluated by assessing serum CK-MB and cTnI levels, myocardial HE and Tunel staining and infarct size. Furthermore, the oxidative stress-related markers including intracellular ROS level, myocardial tissue MDA level, SOD and GSH-PX activities were determined.

**Results:**

Dex postconditioning significantly alleviated myocardial IRI, decreased intracellular ROS and myocardial tissue MDA level, increased SOD and GSH-PX activities. Dex postconditioning significantly up-regulated myocardial expression of Bcl-2, down-regulated Bax and cleaved caspase-3 and decreased cardiomyocyte apoptosis rate. furthermores, Dex postconditioning promoted Nrf2 nuclear translocation, increased myocardial expression of Sirt3 and SOD2 and decreased Ac-SOD2. However, brusatol reversed cardioprotective benefits of Dex postconditioning, significantly decreased Dex-induced Nrf2 nuclear translocation and reduced myocardial expression of Sirt3 and SOD2.

**Conclusions:**

Dex postconditioning can alleviate myocardial IRI by suppressing oxidative stress and apoptosis, and these beneficial effects are at least partly mediated by activating the Nrf2/Sirt3/SOD2 signaling pathway.

## 1. Introduction

Ischemic heart disease, especially acute myocardial infarction (AMI), has become one of the leading causes of morbidity and mortality globally [1]. As restoring the blood flow of ischemic myocardium as soon as possible is generally believed as the most efficient treatment of AMI, numerous clinical intervention strategies such as coronary artery bypass grafting or primary percutaneous coronary intervention have been developed [2,3]. However, restoring blood flow of ischemic myocardium can paradoxically worsen myocardial injury, which is called ischemia/reperfusion injury (IRI) [4]. The available evidence indicates that IRI can even lead up to 50% of final myocardial infarct size [5]. In past decades, numerous studies regarding myocardial IRI have been conducted, but the complex internal pathogenic mechanisms remained to be fully elucidated [6]. It is widely accepted that oxidative stress is a vital factor for the development of myocardial IRI, as it initiates at the onset of reperfusion and triggers subsequent series of pathophysiological processes [7]. Thus, regulating oxidative stress may be one of the promising strategies to attenuate myocardial IRI.

Nuclear factor E2-associated factor 2 (Nrf2), a member of the basic leucine transcription factor family, is one of the crucial antioxidant proteins in the defense system against oxidative stress [8]. Under physiological conditions, Nrf2 is connected to Kelch-like ECH-associated protein 1 (Keap1) and retained in cytoplasm. Once exposure to oxidative stress situations, Nrf2 is released from Keap1 and translocated into the nucleus, in which it binds to antioxidant response elements (ARE) and then regulates the expression of antioxidant genes and controls the transcriptional expression of downstream antioxidant enzymes [8,9]. It has been shown that activation of Nrf2 can protect against myocardial IRI in animal models [10,11]. Sirtuin-3 (Sirt3) belongs to the family of conserved nicotinamide adenine dinucleotide- (NAD^+^-) dependent deacetylases, which is localized in the mitochondrial matrix and can modulate mitochondrial reactive oxygen species (ROS) homeostasis by regulating main antioxidant enzymes such as manganese superoxide dismutase 2 (SOD2) [12]. Actually, Sirt3 can transform acetylated-SOD2 (Ac-SOD2) into SOD2 to eliminate ROS [13]. It has been demonstrated that Nrf2/Sirt3/SOD2 signaling pathway is closely related to oxidative stress. For example, in *in vivo and in vitro* experiments, tert-butylhydroquinone ameliorates radiocontrast-induced nephropathy via activating the Nrf2/Sirt3/SOD2 signaling pathway [14]. In human umbilical cord blood mesenchymal stem cells, 17β-Estradiol protects cells against high glucose-induced mitochondrial ROS production and autophagic cell death through activating the Nrf2/Sirt3/Mn-SOD signaling pathway [15]. However, there has been no study determining whether Nrf2/Sirt3/SOD2 signaling pathway is involved in protective benefits of any treatment against myocardial IRI.

Dexmedetomidine (Dex), a highly selective α2-receptor agonist with characteristics of sedation and analgesia, has been widely applied for surgical and ICU patients [16]. It has been demonstrated that Dex can alleviate the IRI of vital organs including heart by many pathways, such as antioxidation, anti-apoptosis, anti-inflammation and mediating autophagy [17]. Although accumulating evidences have suggested that Dex has strong antioxidative properties and Nrf2/Sirt3/SOD2 signaling pathway plays an important role in intrinsic antioxidative mechanisms, the relationship between Dex and Nrf2/Sirt3/SOD2 signaling pathway in protective effects of Dex against myocardial IRI remained elusive. A recent study demonstrated that Dex treatment could attenuate cerebral ischemic injury by promoting the polarization of M2 microglia via the Nrf2/HO-1/NLRP3 pathway in mouse model of middle cerebral artery occlusion and *in vitro* microglia subjected to oxygen-glucose deprivation treatment [18]. Similarly, another study reported that Dex alleviated hepatic injury via the inhibition of oxidative stress and activation of the Nrf2/HO-1 signaling pathway in a model of glucose deprivation/reoxygenation (OGD/R)-induced WRL-68 cells injury [19]. Recently, several literatures have shown that Dex could attenuate myocardial IRI and hypoxia-induced cardiomyocyte injury by via activation of Nrf2-related signaling pathways [20,21]. These studies provide further evidence that there may be a strong association between myocardial protection of Dex treatment and activation of Nrf2. However, Dex treatment was mainly implemented before myocardial ischemia or cardiomyocyte hypoxia in previous studies [20–23], i.e. preconditioning intervention. As the occurrence of acute myocardial ischemia is usually unpredictable in the real world, the clinical value of preconditioning intervention is very limited. It is certainly that postconditioning regimen is more consistent with clinical practice and has a greater clinical value. Unfortunately, there have been only a few studies assessing the protective effects of Dex postconditioning against myocardial IRI, and the inconsistent results are obtained [24,25]. Consequently, this experiment was designed to determine whether Dex postconditioning could provide a protection against myocardial IRI and to explore whether Nrf2/Sirt3/SOD2 signaling pathway was involved in protection of Dex postconditioning against myocardial IRI *in vivo*.

## 2. Materials and methods

### 2.1. Animals

After the experimental protocols were approved by the Committee on the Ethics of Animal Experiments of Beijing Friendship Hospital (Ethics No. 21-1003), Male Sprague–Dawley rats, aged seven to eight weeks old and weighing about 230–270 g, were purchased from Beijing Vital River Laboratory Animal Technology Co., Ltd. (Beijing, China). The animals were housed in a specific pathogen-free laboratory at the Animal Experimental Center of Beijing Friendship Hospital under standard conditions with controlled temperature (24 ± 2°C), humidity (40–60%) and a 12 h light/dark cycle. After the animal acclimated to the housing environment for seven days, experiment was carried out following the Guides for the Care and Use of Laboratory Animals published by the United States National Institutes of Health.

### 2.2. Establishment of *in vivo* myocardial IRI model

The *in vivo* model of myocardial IRI was built as previously described [26]. In brief, the rats were fasted for 12 h with free access to water and then anesthetized by intraperitoneal injection of 10 ml/kg 2% 2,2,2-tribromoethanol (TBE, Avertin, Sigma, St. Louis, MO, USA). After tracheotomy, the tracheal cannula was connected to a small animal ventilator (HX-101E, Chengdu Techman Instrument Co., Ltd., Chengdu, China). The ventilation rate was adjusted to 60–80 breaths/min, with tidal volume of 2–3 ml/100 g body weight and inspiratory/expiratory ratio of 1:1. The lead II electrocardiogram (ECG) was continuously recorded by the means of needle electrodes placed subcutaneously on the limbs. By the right common carotid artery cannula, the hemodynamic variables were continuously monitored with a transducer (BL-420N, Chengdu Techman Instrument Co., Ltd., Chengdu, China). Following the rat heart was exposed through a left thoracotomy in the fourth intercostal space, myocardial ischemia was achieved by a ligation of the left anterior descending (LAD) coronary artery. After a 30-min occlusion of LAD, a 120-min reperfusion was performed. The criteria for successful preparation of the myocardial IRI model were as follows [26]: with ischemia, myocardial tissues were seen as pale and cyanotic along with obvious ST segment elevation on ECG, whereas after reperfusion, myocardial tissues in the ischemic region turned red, and the ST segment elevation occurring within ischemic period significantly decreased.

### 2.3. Treatment protocols

**Figure 1.**
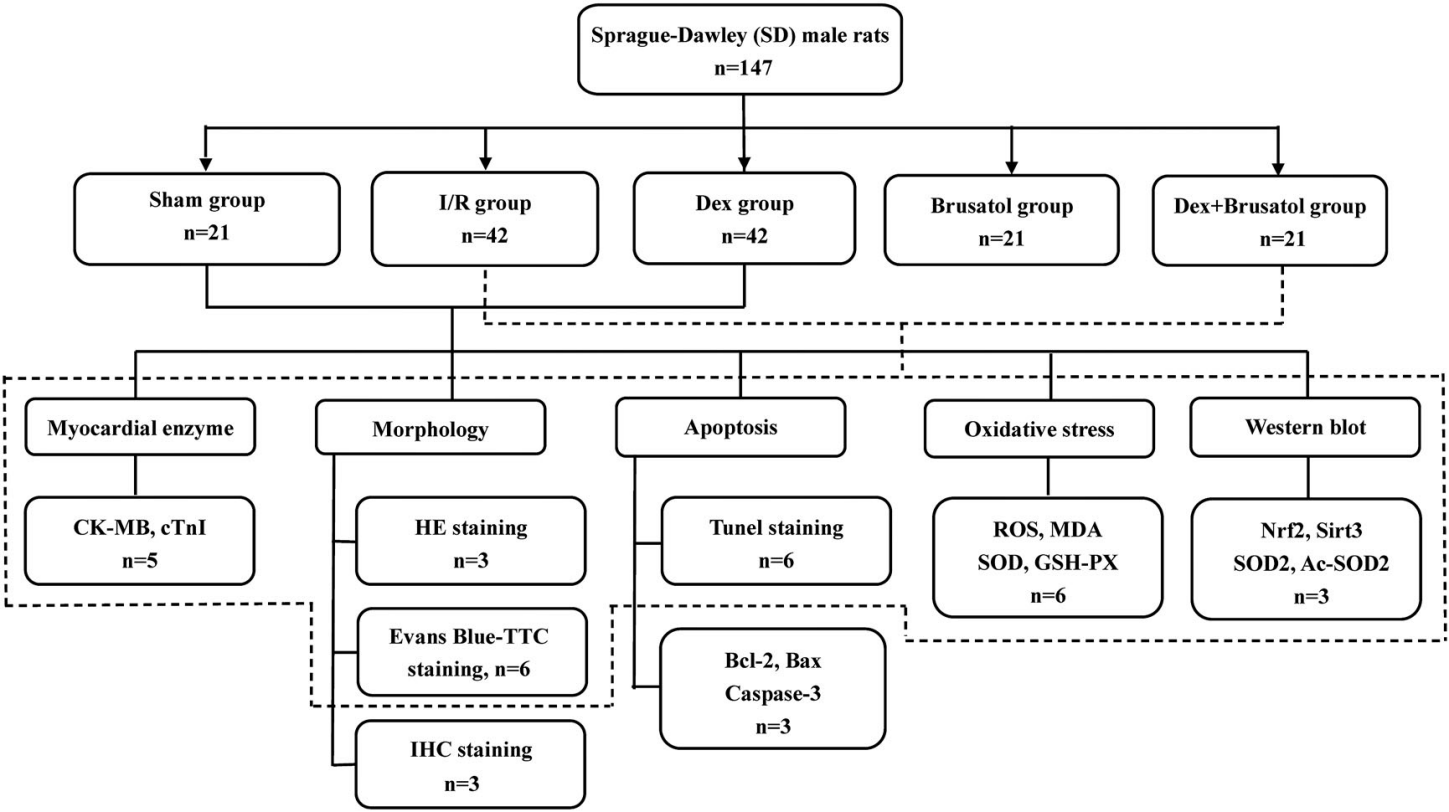
Flow chart of the study. The solid lines represent the experimental sections in Figure 2–4. The dashed lines represent the experimental sections in Figures 5 and 6. The number of rats shown in the flow chart is the final number included in each group. The specific allocation scheme is detailed in methods of the manuscript.

A 22G intravenous catheter was inserted into the right jugular vein for intravenous infusion of Dex (Heng Rui Pharmaceuticals Co., Ltd, Nanjing, Jiangsu, China) or normal saline. By a random number table, 147 rats were randomly divided into five groups receiving different treatments: (1) Sham group (*n* = 21), animal received the sham treatments that LAD was threaded but not ligated for 150 min; (2) ischemia/reperfusion (I/R) group (*n* = 42), animal received the I/R procedures with a 30-min ischemia followed by a 120-min reperfusion; (3) Dex group (*n* = 42), animal received the I/R procedures and intravenous Dex, which was initialed at 5 min before reperfusion and lasted for 25 min (6 μg/kg/h × 10 min + 0.7 μg/kg/h × 15 min) [27]. Dex was freshly dissolved in normal saline with a concentration of 0.5 μg/ml; (4) Brusatol group (*n* = 21): Brusatol (MedChemExpress, Monmouth Junction, NJ, USA), a Nrf2 antagonist, was given intraperitoneally according to a dose of 0.4 mg/kg, which was started 10 days before I/R procedures and executed every other day, with a total of five doses for each animal [28]. Then animal received the I/R procedures; (5) Dex + Brusatol group (*n* = 21), animal received the five times of Brusatol treatment before I/R procedures and then I/R procedures and Dex treatments were carried out. Other than Dex group, animals in other groups received intravenous infusion of normal saline, which was initialed at 5 min before reperfusion and lasted for 25 min, with the same infusion rate of intravenous Dex. The flow chart was shown in Figure 1.

### 2.4. Serum creatine kinase isoenzyme (CK-MB) and cardiac troponin I (cTnI) assessments

At the end of reperfusion, five rats were randomly selected from each group and 4 ml of blood sample was collected from the right common carotid artery in each rat. Following 30 min of concentration at room temperature, the clear serum supernatant was collected through centrifugation at 3000 rpm/min at 4°C for 15 min, and then frozen at a −80°C condition for further analysis. Serum myocardial enzyme levels were quantified, respectively, using CK-MB and cTnI ELISA kits (Elabscience, Wuhan, China), according to the manufacturer’s instructions.

### 2.5. Malondialdehyde (MDA), superoxide dismutase (SOD) and glutathione peroxidase detection (GSH-PX)

At the end of reperfusion, six rats were randomly selected from each group and were euthanized by increasing depth of anesthesia and intravenous injection of 10% potassium chloride 100 mg/kg. Then the heart was cut off from the root of aorta and immediately placed in the precooled PBS at 4°C to rinse the blood. The levels of MDA and GSH-PX activity in the lysates of ischemic myocardium were, respectively, detected by MDA assay kits (Nanjing Jiancheng Bioengineering Institute, Nanjing, China) and GSH-PX assay kits (Nanjing Jiancheng Bioengineering Institute, Nanjing, China), according to the manufacturer’s protocol. The SOD enzymatic activity in ischemic myocardium was measured with Cu/Zn-SOD and Mn-SOD assay kits with WST-8, according to the manufacturer’s instructions (Beyotime Institute of Biotechnology, Shanghai, China).

### 2.6. ROS level measurement

As described above, after reperfusion and euthanasia, six rats were randomly selected from each group for tissue sections. The myocardial tissue in ischemic area was collected for paraffin section (Used for HE, IHC and Tunel staining) and cryosection to detect ROS. ROS generation in ischemic myocardium was measured using the oxidant-sensitive fluorogenic probe dihydroethidium (DHE) (Sigma-Aldrich, St. Louis, MO, USA) as previously described [29]. Fresh and frozen left ventricular tissues (10 μm sections) were incubated with 10 μmol/l DHE in PBS in the dark for 30 min at 37°C. The level of red fluorescence apparent is directly proportional to the intracellular ROS level. The fluorescence intensity of intracellular ROS was recorded using a fluorescence microscope (Olympus IX81, Tokyo, Japan). The average fluorescence intensity was assessed with the Image J software program (version 1.46, NIH, Bethesda, MD, USA).

### 2.7. Myocardial histopathology and immunohistochemistry staining

The staining of hematoxylin–eosin (HE) was performed on 4 μm sections of ischemic myocardial tissues cut from the 4% paraformaldehyde solution-fixed, paraffin-embedded blocks [24]. The tissue sections were observed and photographed by a light microscope (DM2500, Leica, Wetzla, Hessen, Germany) to evaluate the extent of pathological changes in the ischemic myocardium (*n* = 3 in each group). For immunohistochemistry (IHC) staining [14], antigens were retrieved by the microwave antigen retrieval method using citric acid buffer and detected via SP-9000 staining (Golden Bridge Biotechnology, Beijing, China). The tissue sections were incubated with rabbit polyclonal anti-Nrf2 (1:200, Proteintech Group, Inc. Wuhan, China) and rabbit polyclonal anti-Sirt3 (1:200, Cell Signaling Technology, Danvers, MA, USA) antibodies overnight at 4°C, followed by incubation with the goat anti-rabbit secondary antibody (1:100; Golden Bridge Biotechnology, Beijing, China) for 20 min. After the reaction was terminated, the tissue sections were observed under a light microscope (*n* = 3 in each group).

### 2.8. Terminal deoxynucleotidyl transferase-mediated deoxyuridine triphosphate nick end-labeling (Tunel) staining

After 4 μm tissues sections were cut from the 4% paraformaldehyde solution-fixed, paraffin-embedded blocks, the Tunel staining was performed using the In Situ Cell Death Detection kit, Fluorescein (Roche Diagnostics Deutschland GmbH, Mannheim, Baden-Wurttemberg, Germany), following the manufacturer’s protocols [24]. The apoptotic cells were observed by a laser scanning confocal microscope (Olympus IX81, Tokyo, Japan). The Tunel positive nucleus was represented by green fluorescence. The number of apoptotic cells was counted by Image J software (version 1.46, NIH, Bethesda, MD, USA). Three view fields were selected for each section, with at least 200 cells present in each field. Apoptotic index = number of positive cells/total cells × 100% [30] (*n* = 6 in each group).

### 2.9. Infarct size assessment by Evans blue and 2,3,5-triphenyltetrazolium chloride staining

At the end of reperfusion, six rats were randomly selected from each group, the LAD was reoccluded and 1 ml of 2% Evans blue was injected through the right common carotid artery to stain the normally perfused region of heart and delineate the area at risk (AAR). After the body was stained blue, the rat was euthanized by increasing depth of anesthesia and intravenous injection of 10% potassium chloride 100 mg/kg. Then the heart was excised and quickly frozen at −80°C for 15 min. Thereafter, the frozen hearts were sliced perpendicularly along the long axis into 1 mm sections below the ligation position of the LAD, followed by incubation for 15 min in 1% 2,3,5-triphenyltetrazolium chloride at 37°C in the dark to determine the infarcted myocardium. The sections were subsequently fixed by 10% formalin overnight and photographed using a digital camera. The five sections were selected from each rat and observed. Analysis of the sections using the Image J software (version 1.46, NIH, Bethesda, MD, USA) by a blinded investigator allowed for assessment of the AAR and infarct size, which was expressed as a percentage of the AAR [11] (*n* = 6 in each group).

### 2.10. Western blot analysis

After reperfusion and euthanasia, three rats were randomly selected from each group for western blotting. The heart was cut off from the root of aorta and immediately placed in the precooled PBS at 4°C to rinse the blood. After myocardial tissues in the ischemic area were taken, the total protein of myocardium was extracted with RIPA buffer (Solarbio, Beijing, China) and the nuclear extraction was prepared using a NE-PER Nuclear Cytoplasmic Extraction Reagent kit (Pierce, Rockford, IL, USA). Protein concentration was measured with bicinchoninic acid (BCA) kits (Solarbio, Beijing, China). The extracted proteins were separated by 12% polyacrylamide gel electrophoresis and transferred into the polyvinylidene fluoride (PVDF) membranes (Merck KGaA, Darmstadt, Germany). After blocked by 5% skim milk powder at room temperature for 1 h, the membranes were appended with primary antibodies Nrf2 (1:1000, Abcam, Cambridge, MA, USA), Sirt3 (1:1000, Abcam, Cambridge, MA, USA), SOD2 (1:1000, Abcam, Cambridge, MA, USA), Ac-SOD2 (1:1000, Abcam, Cambridge, MA, USA), Bcl-2 (1:1000, Abcam, Cambridge, MA, USA), Bax (1:2000, Abcam, Cambridge, MA, USA), Caspase-3 (1:1000, Abcam, Cambridge, MA, USA), β-actin (1:1000, Cell Signaling Technology, Danvers, MA, USA) and Histone H3 (1:1000, Cell Signaling Technology, Danvers, MA, USA) at 4°C overnight. Then the PVDF membranes were cleaned and incubated with horseradish peroxidase-conjugated second antibody (1:2000, Abcam, Cambridge, MA, USA) for 2 h at room temperature. The target bands were observed by the Immobilon Western chemiluminescent HRP substrate (EMD Millipore Corporation, Burlington, USA) and quantified by Image J software (version 1.46, NIH, Bethesda, MD, USA). Protein expression was calculated with β-actin and Histone H3 as the internal control. All experiments were repeated in triplicates.

### 2.11. Statistical analysis

The SPSS 17.0 statistical software (SPSS Inc., Chicago, IL, USA) was used for data analysis. The Kolmogorov–Smirnov test was used to determine the normality of distribution for all parametric data. Furthermore, the Levene median test was adopted to evaluate the homogeneity of variance for the parametric data. If the data were normally distributed and had homogeneous variance, they were expressed as a mean ± standard error of the mean (SEM). One-way analysis of variance (ANOVA) was used for intergroup comparisons. Repeated-measures ANOVA was applied for within-group comparisons. The Tukey’s multiple comparison tests were used for multiple post hoc comparisons. When the data were not normally distributed or had inhomogeneous variance, they were expressed as median (interquartile range) and were compared using the Kruskal–Wallis and Mann–Whitney *U*-tests. The GraphPad Prism for Windows (Version 9, GraphPad Software Inc., San Diego, CA, USA) was used for production of figures. A *P*-value of less than .05 was considered statistically significant.

## 3. Results

### 3.1. Dex decreased serum myocardial enzyme levels, ameliorated myocardial histological changes and limited infarct size

**Figure 2.**
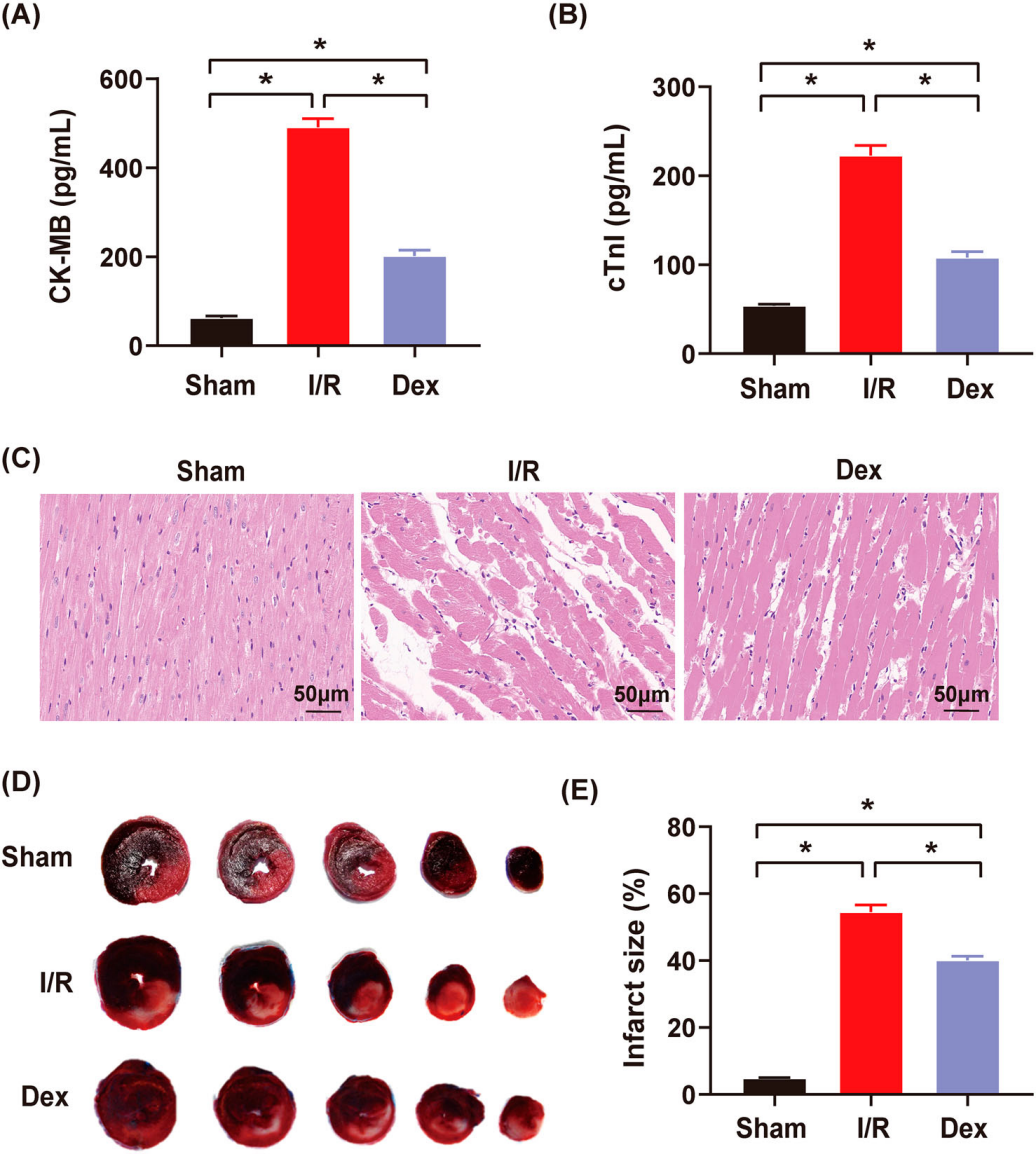
The effects of Dex postconditioning on myocardial enzymes, pathological changes of myocardial damages and infarct sizes in the rats with myocardial IRI. (A) and (B) Serum CK-MB and cTnI levels (*n* = 5); (C) Pathological changes of myocardial damages with HE staining (*n* = 3), magnification: ×200, bar = 50 μm; (D) and (E) Infarct sizes determined by the Evans blue and TTC staining (*n* = 6). Date was expressed as the mean ± SEM. *P *< *.05.

Compared to the Sham group, serum CK-MB and cTnI levels were significantly increased in the I/R and Dex groups (Figure 2(A,B), *P* < .05). Compared to the I/R group, serum CK-MB and cTnI levels were significantly decreased in the Dex group (*P* < .05). As shown in Figure 2(C), myocardial tissues with the HE staining had normal histological structures, smooth mesenchyme and regular muscle bundles in the Sham group. However, myocardial tissues had irregular muscle bundles, massive ruptured muscle fibers and interstitial edema in the I/R group. These pathological changes of myocardial tissues induced by I/R intervention were significantly alleviated in the Dex group. The infarct size was significantly increased in the I/R and Dex groups compared to the Sham group (*P* < .05), but it was significantly reduced in the Dex group compared to the I/R group (Figure 2(D,E), *P* < .05).

### 3.2. Dex alleviated myocardial oxidative stress and apoptosis by I/R intervention

**Figure 3.**
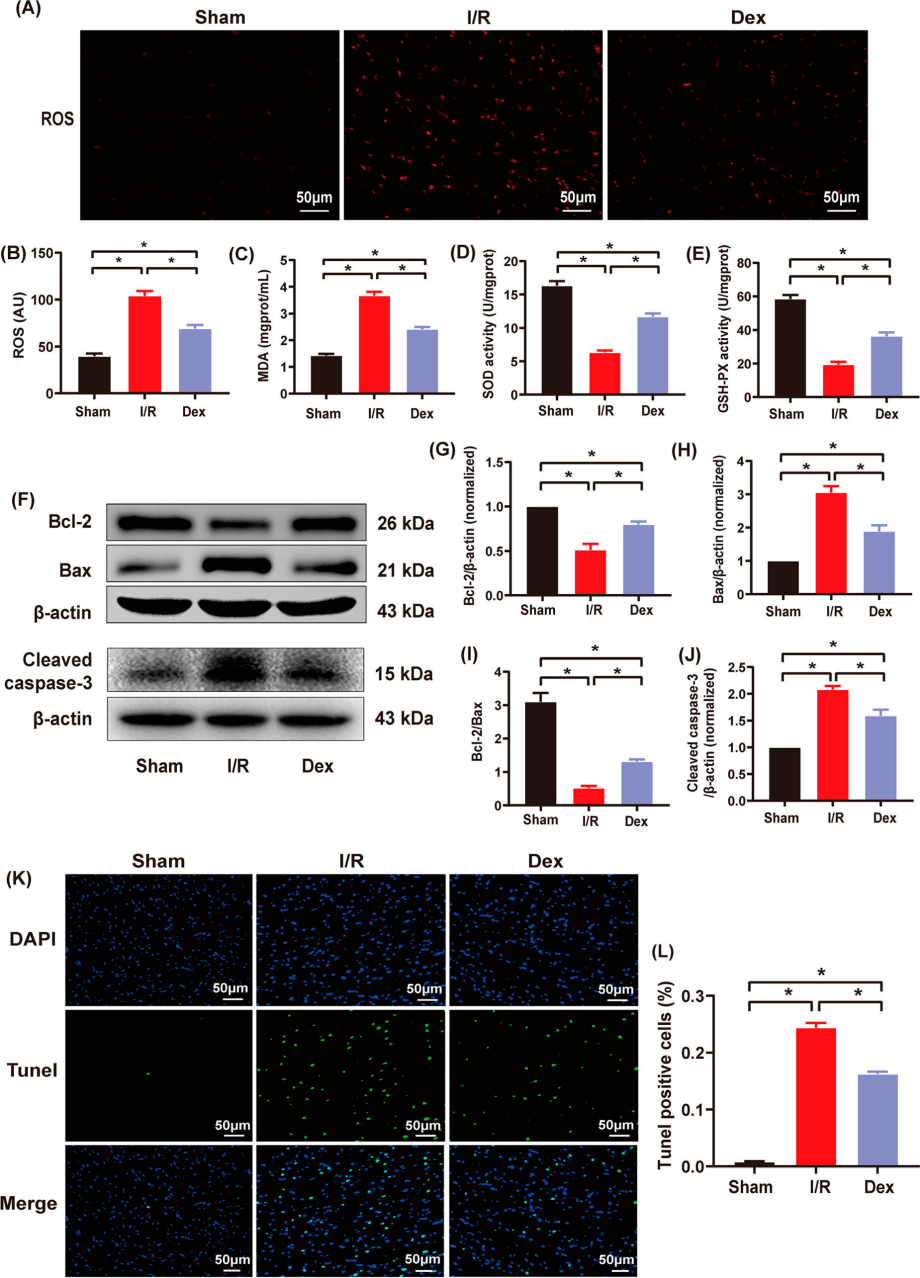
The effects of Dex postconditioning on oxidative stress and cardiomyocyte apoptosis in the rats with myocardial IRI. (A) and (B) Immunofluorescence of ROS (*n* = 6), magnification: ×200, bar = 50 μm; (C) Myocardial MDA level (*n* = 6). (D) and (E) Myocardial SOD and GSH-PX activities (*n* = 6); (F) Myocardial expression of Bcl-2, Bax and cleaved caspase-3 measured by western blotting (*n* = 3); (G–J) Quantitative analyses of myocardial Bcl-2, Bax, Bcl-2/Bax and cleaved caspase-3; (K) and (L) Cardiomyocyte apoptosis rates assessed by the Tunel assay (*n* = 6). Magnification, ×200, bar = 50 μm. Date was expressed as the mean ± SEM. *P *< *.05.

The intracellular ROS fluorescence and myocardial MDA level were significantly increased in the I/R group compared to the Sham group, but they were significantly reduced in the Dex group compared to the I/R group (Figure 3(A–C), *P* < .05). The myocardial SOD and GSH-PX activities were significantly decreased in the I/R group compared to the Sham group (*P* < .05), but they were markedly increased in the Dex group compared to the I/R group (Figure 3(D,E), *P* < .05).

As shown in Figure 3(F–J), I/R intervention down-regulated myocardial expression of anti-apoptotic factor Bcl-2 and up-regulated myocardial expression of pro-apoptotic factors such as Bax and cleaved caspase-3 (*P* < .05). However, Dex treatment reversed these changes of apoptosis-related factors induced by I/R intervention. The cardiomyocyte apoptosis rate was significantly increased in the I/R and Dex groups compared to the Sham group (*P* < .05), but it was significantly decreased in the Dex group compared to the I/R group (Figure 3(K,L), *P* < .05).

### 3.3. Dex enhanced Nrf2 nuclear translocation, and increased myocardial expression of Sirt3 and SOD2

**Figure 4.**
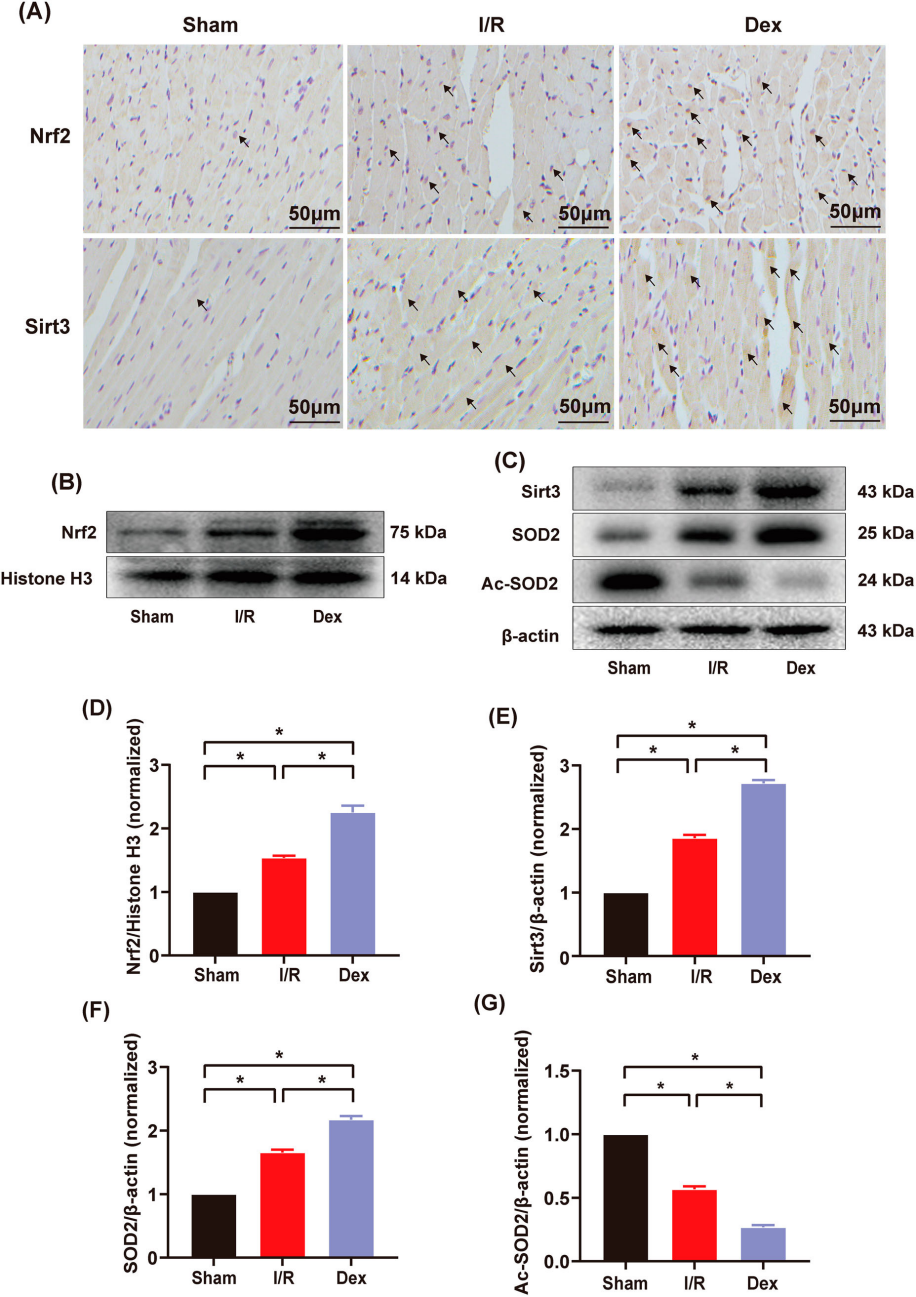
Immunohistochemical staining, western blotting and quantitative analysis of protein expression of myocardial Nrf2/Sirt3/SOD2 signaling pathway. (A) Immunohistochemical staining of myocardial Nrf2 and Sirt3 (*n* = 3), arrows indicate positive results; magnification: ×200, bar = 50 μm; (B) and (C) The expression of myocardial nuclear-Nrf2, Sirt3, SOD2 and Ac-SOD2 measured by western blotting (*n* = 3); (D–G) Quantitative analyses of myocardial nuclear-Nrf2, Sirt3, SOD2 and Ac-SOD2. Date was expressed as the mean ± SEM. *P *< *.05.

As shown in Figure 4(A–E), both nuclear translocation of Nrf2 and myocardial expression of Sirt3 were significantly elevated in the I/R group compared to the Sham group (*P* < .05). Moreover, both variables were significantly higher in the Dex group than in the I/R group (*P* < .05).

The myocardial expression of SOD2 was significantly increased and myocardial expression of Ac-SOD2 was decreased in the I/R group compared to the Sham group (Figure 4(C,F,G), *P* < .05). The myocardial expression of SOD2 was significantly increased and myocardial expression of Ac-SOD2 was significantly decreased in the Dex group compared to the I/R group (*P* < .05).

### 3.4. Brusatol attenuated cardioprotective benefits of Dex against myocardial IRI

**Figure 5.**
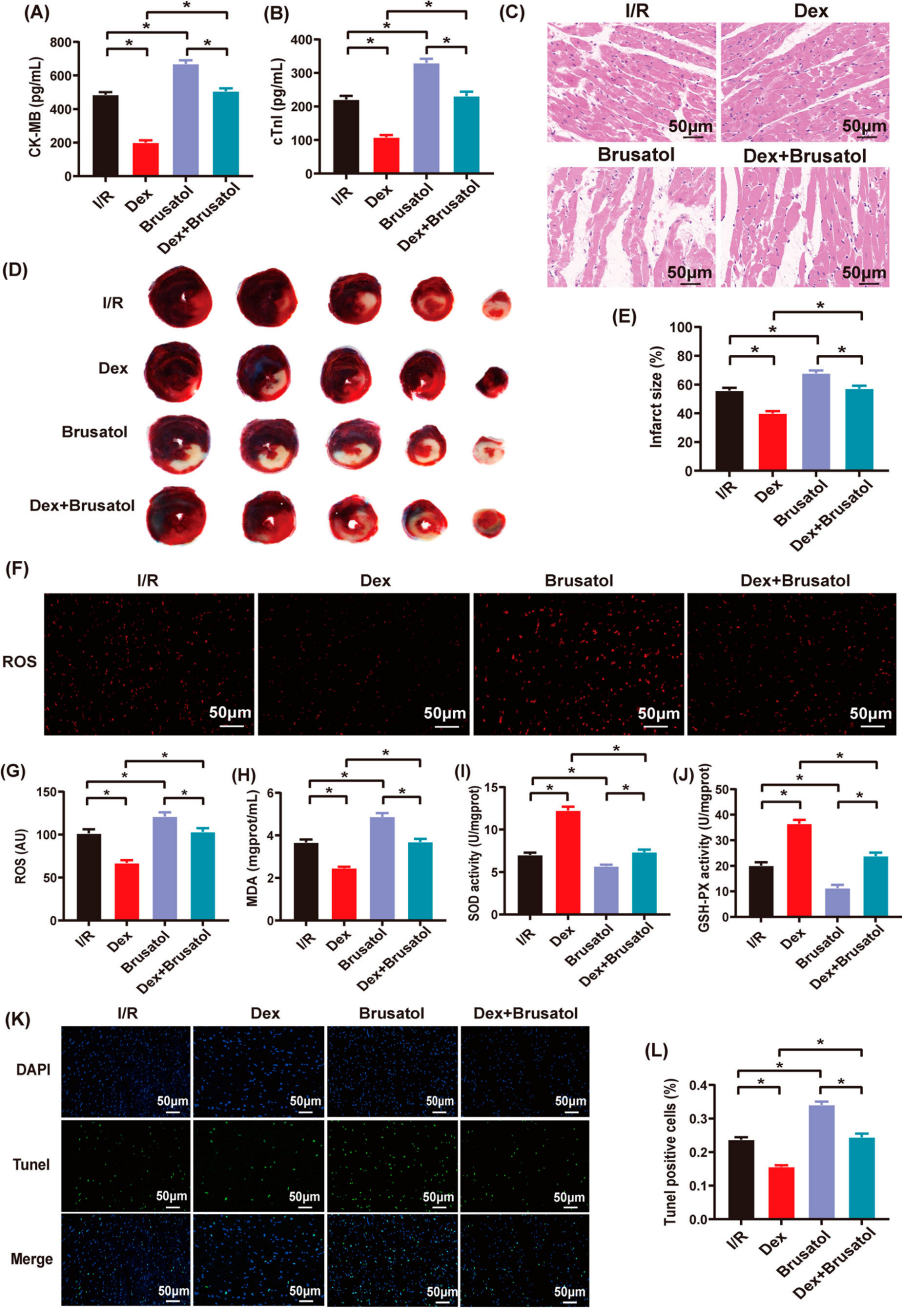
The effects of Brusatol administration on the protection of Dex postconditioning against myocardial IRI. (A) and (B) Serum CK-MB and cTnI levels (*n* = 5); (C) Pathological changes of myocardial damages observed by HE staining (*n* = 3), magnification, ×200, bar = 50 μm; (D) and (E) Infarct sizes determined by the Evans blue and TTC staining (*n* = 6); (F) and (G) Immunofluorescence of ROS (*n* = 6), magnification: ×200, bar = 50 μm; (H) Myocardial MDA level (*n* = 6); (I) and (J) Myocardial SOD and GSH-PX activities (*n* = 6); (K) and (L) Cardiomyocyte apoptosis assessed by the Tunel assay (*n* = 6), magnification: ×200, bar = 50 μm. Date was expressed as the mean ± SEM. *P *< *.05.

As shown in Figure 5(A–E), serum CK-MB and cTnI levels, myocardial pathological changes and infarct size in the I/R group were similar to described above. However, these changes of myocardial damage were significantly alleviated in the Dex group. Furthermore, changes of serum CK-MB and cTnI levels, myocardial pathological features and infarct size all became more severe in the Brusatol group compared to both I/R and Dex groups (*P* < .05). However, serum CK-MB and cTnI levels, myocardial pathological features and infarct size showed no difference between I/R and Dex + Brusatol groups (*P* > .05).

Compared to the I/R and Dex groups, intracellular ROS fluorescence and myocardial MDA level were significantly increased in the Brusatol group (Figure 5(F–H), *P* < .05). Meanwhile, myocardial SOD and GSH-PX activities were significantly decreased in the Brusatol group compared to the I/R and Dex groups (Figure 5(I,J), *P* < .05). The cardiomyocyte apoptosis rate was significantly increased in the Brusatol group compared to both I/R and Dex groups (Figure 5(K,L), *P* < .05). However, intracellular ROS fluorescence, myocardial MDA level, myocardial SOD and GSH-PX activities, and cardiomyocyte apoptosis rate were comparable between I/R and Dex + Brusatol group (Figure 5(F–L), *P* > .05).

### 3.5. Dex alleviated myocardial IRI by activating the Nrf2/Sirt3/SOD2 signaling pathway

**Figure 6.**
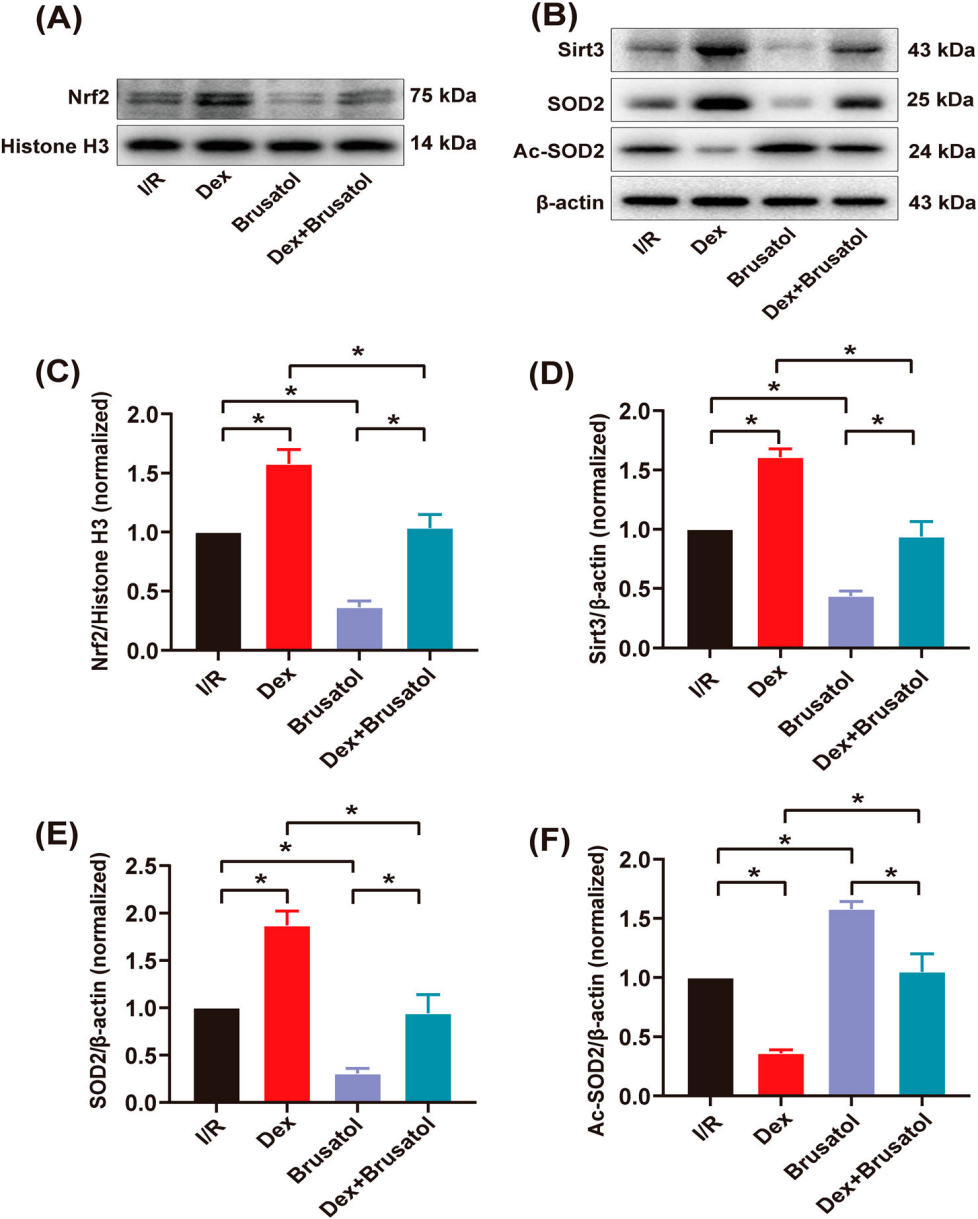
Western blotting and quantitative analysis of protein expression of myocardial Nrf2/Sirt3/SOD2 signaling pathway. (A) and (B) The expression of myocardial nuclear-Nrf2, Sirt3, SOD2 and Ac-SOD2 measured by western blotting (*n* = 3); (C)–(F) Quantitative analyses of myocardial nuclear-Nrf2, Sirt3, SOD2 and Ac-SOD2. Date was expressed as the mean ± SEM. *P *< *.05.

As compared to the I/R intervention, Brusatol treatment significantly restricted the nuclear translocation of Nrf2, decreased myocardial expression of Sirt3 and SOD2, and increased myocardial expression of Ac-SOD2 (Figure 6(A–F), *P* < .05). Furthermore, Brusatol treatment partially reversed myocardial expression changes of nuclear-Nrf2, Sirt3, SOD2 and Ac-SOD2 by Dex treatment (*P* < .05).

## 4. Discussion

This experiment was designed to evaluate the protective effects of Dex postconditioning against *in vivo* myocardial IRI and to determine whether the Nrf2/Sirt3/SOD2 signaling pathway was involved in cardioprotective effects of Dex postconditioning. The main results of this experiment included: (1) Dex postconditioning significantly reduced serum levels of myocardial injury biomarkers, alleviated pathological features of myocardial damage and limited infarct size; (2) Dex postconditioning significantly decreased intracellular ROS and myocardial MDA level, restored myocardial SOD and GSH-PX activities, up-regulated myocardial expression of anti-apoptotic factor Bcl-2 and down-regulated myocardial expression of pro-apoptotic factors such as Bax and cleaved caspase-3, and significantly decreased cardiomyocyte apoptosis rate; (3) Nrf2 antagonist, Brusatol, largely abolished cardioprotective benefits of Dex postconditioning, and down-regulated myocardial expression of Sirt3 and SOD2. All of these indicate that Dex postconditioning ameliorates myocardial IRI by antioxidation and anti-apoptosis, which is at least partly contributed to activation of the Nrf2/Sirt3/SOD2 signaling pathway. Thus, this experiment validates our hypothesis.

Myocardial IRI is a complicated pathophysiological process which is associated with a series of intracellular abnormal changes, such as excessive oxidative or endoplasmic reticulum stress and inflammatory response, acidosis, calcium overload, energy metabolism disorders, enhanced apoptosis or autophagy and others [31,32]. Although various ischemic conditionings and pharmacological interventions have been attempted to ameliorate myocardial IRI, the translation from experimental findings to clinical application is extremely unsatisfactory, and the mortality of patients with AMI remains significant [33,34]. Thus, the development of new cardioprotective strategies to attenuate myocardial IRI and improve the survival rate and quality of life of patients with AMI is urgently needed.

Dex is an α2-adrenoceptor agonist with sedative, and analgesic and anti-sympathetic effects [16]. Both *in vitro* and *in vivo* experiments have demonstrated the protective benefits of Dex preconditioning against the IRI of vital organs including brain, heart, liver, lung and kidney [35–39]. However, there have been only a few studies determining the effects of Dex postconditioning on myocardial IRI and inconsistent results are obtained. For example, recent work of Peng et al. [27] in the myocardial IRI rat model and cardiomyocyte H/R model reveals that cardioprotection of Dex postconditioning is partly mediated via the inhibition of apoptosis by targeting HIF-1α signal pathway. In line with these findings, our experiment showed that Dex postconditioning exerted powerful antioxidation and anti-apoptosis effects and provided a significant protection against myocardial IRI. However, the study of Mimuro et al. [25] in the IRI model of isolated rat hearts produced the different findings, i.e. Dex administration at the initiation of reperfusion increased the infarct size. This inconsistent finding may mainly be attributable to the application of isolated rat heart model in the experiment.

Nrf2, a crucial transcription factor, can transfer into nucleus and up-regulate expression of antioxidant enzymes once cells exposure to oxidative stress conditions [8]. It has been reported that genetical and pharmacological activation of Nrf2 can attenuate ischemic myocardial injury in animal models, while loss of Nrf2 exacerbates heart failure following myocardial infarction [40,41]. In fact, in response to myocardial IRI, the activation and nuclear translocation of Nrf2 are increased, which represents a cellular self-protective mechanism [42]. Zeng et al. [10] demonstrated that dihydrotanshinone-I could regulate PKC-δ and PKB/GSK-3β/Fyn signaling to promote Nrf2 nuclear accumulation and exert a protection against myocardial IRI in both *in vivo* and *in vitro* experiments. Consistently, the results of immunohistochemistry staining and western blotting in our experiment showed that Nrf2 nuclear translocation was increased by myocardial IRI. Collectively, these findings highlight the importance of Nrf2 nuclear translocation in the development of myocardial IRI and suggest that the application of Nrf2-inducer may be an essential strategy to attenuate myocardial IRI. Furthermore, bioinformatic analysis and experimental validation in cell culture demonstrate that Nrf2 is a transcriptional regulator of Sirt3 expression [9]. Sirt3 is highly expressed in the myocardium and is considered as the most important deacetylase [43]. Existing evidence indicates that more than 65% of all mitochondrial proteins are acetylated, and Sirt3 acts as the primary deacetylase for these proteins [44]. Zhai et al. [45] demonstrate that melatonin treatment significantly inhibits I/R-induced cardiac dysfunction, oxidative stress and apoptosis by activating the Sirt3/SOD2 signaling pathway in *in vivo and in vitro* experiments. However, these effects of melatonin can be significantly abolished by selective Sirt3 inhibitor and Sirt3-targeted siRNA. All of these indicate that Nrf2 or Sirt3 plays an indispensable role in antioxidation. In addition, as described above, oxidative stress is closely related to the Nrf2/Sirt3/SOD2 signaling pathway [14,15].

This study is the first to determine whether Dex postconditioning provides a protection against myocardial IRI by activating Nrf2/Sirt3/SOD2 signaling pathway. The results showed that Dex treatment significantly strengthened Nrf2 nuclear translocation, facilitated myocardial expression of Sirt3 and SOD2, and attenuated myocardial injury. To further test our hypothesis, Brusatol, a potent Nrf2 inhibitor, was used to selectively reduce myocardial expression of Nrf2 by enhancing ubiquitination and degradation [46]. Our findings showed that when only Brusatol was used, Nrf2 nuclear translocation was significantly decreased and severity of myocardial IRI was aggravated. These findings are in accordant with the result of Lin et al.’ study, in which Brusatol deteriorates lung IRI in mice model [47]. Most important, our results showed that Brusatol administration before I/R intervention significantly decreased Dex-induced Nrf2 nuclear translocation, decreased myocardial expression of Sirt3 and SOD2, and reversed protection of Dex postconditioning against myocardial IRI. These results are also consistent with the findings of Lin et al’ study, in which Brusatol reduces the expression of Nrf2 and block antioxidant and anti-pyroptotic effects of recombinant HMGB1 in mice model of lung IRI [47]. Besides, Gu et al. [48] demonstrated that pinocembrin could inhibit cardiomyocyte pyroptosis against doxorubicin-induced cardiac dysfunction via regulating Nrf2/Sirt3 signaling pathway both *in vitro* and *in vivo* experiments. All of these results from previous and our experiments support that Nrf2 plays a crucial role of the development of myocardial IRI and the potential molecular mechanism of Dex postconditioning against myocardial IRI. Accordingly, we consider that Dex postconditioning attenuates myocardial IRI by activating the Nrf2/Sirt3/SOD2 signaling pathway.

There are several limitations in the design of this experiment that deserve special attention. First, this experiment was conducted in the normal rat model of myocardial IRI. It is reported that cardiovascular comorbidities including diabetes mellitus, hypertension, hyperlipidemia and obesity can abolish or attenuate the benefits of cardioprotective interventions against myocardial IRI [49,50]. Thus, our results cannot be generalized to the animals subjected to myocardial IRI with any cardiovascular comorbidity. Second, only a single dose and an intervention time point were designed. Thus, this experiment cannot determine whether cardioprotective effects of Dex postconditioning are the dose-dependent and implementation time of intervention can significantly affect protective effects of Dex postconditioning against myocardial IRI. Third, only oxidative stress, apoptosis and the Nrf2/Sirt3/SOD2 signaling pathway were focused in this experiment. Available evidence indicates that inflammatory inhibition, mitochondrial MCT1 and PI3K/Akt signaling pathway are also involved in the protection of Dex against myocardial IRI [51,52]. Evidently, this study cannot provide any clues for possible contributions of these factors to protection of Dex postconditioning against myocardial IRI. Last, this is a preclinical research in animals and the findings of this experiment cannot be directly extrapolated into clinical practice. Thus, more basic and clinical studies are still required to address the above issues and confirm the protection of Dex postconditioning against myocardial IRI.

## 5. Conclusions

Our results demonstrate that in an *in vivo* rat model of myocardial IRI, Dex postconditioning provides significant protective effects by alleviating oxidative stress and apoptosis. Furthermore, these beneficial effects are at least partly mediated by activating the Nrf2/Sirt3/SOD2 signaling pathway.

## Data Availability

The data used to support the findings of this study are available from the corresponding authors upon request.
